# Dark Tourists: Profile, Practices, Motivations and Wellbeing

**DOI:** 10.3390/ijerph191912100

**Published:** 2022-09-24

**Authors:** José Magano, José A. Fraiz-Brea, Ângela Leite

**Affiliations:** 1 Research Center in Business and Economics (CICEE), Universidade Autónoma de Lisboa, Rua Sta. Marta 47, 5.º Andar, 1150-293 Lisboa, Portugal; 2 ISCET-Higher Institute of Business Sciences and Tourism, Rua de Cedofeita, 285, 4050-180 Porto, Portugal; 3 Department of Business Organization, Business Administration and Tourism Faculty, University of Vigo, 32004 Ourense, Spain; 4 Center for Philosophical and Humanistic Studies, Faculty of Philosophy and Social Sciences, Portuguese Catholic University, Rua de Camões 60, 4710-362 Braga, Portugal

**Keywords:** dark tourism, motivations, practices, dark tourist, tourist profile, tourism wellbeing

## Abstract

This work aims to address whether knowing what dark tourism is (or not) impacts rumination on sadness, self-hatred, hostility, psychological vulnerability, and tourist wellbeing, as well as practices and motivations for dark tourism. A quantitative approach, based on a survey of 993 respondents, reveals that women and more educated participants know more about dark tourism; people who know what dark tourism is have visited more Holocaust museums, sites of human tragedy and natural disasters, concentration camps, and prisons; show more curiosity, need to learn and understand, and need to see morbid things. A model was found showing that gender, age, know/do not know dark tourism, and motivations (curiosity, the need to learn, the need to understand, and pleasure) explained 38.1% of a dark tourism practice index. Most findings also indicate that rumination on sadness, self-hatred, hostility, and psychological vulnerability are associated with darker practices. Greater wellbeing was not found in participants who knew in advance what dark tourism was. Interestingly, participants who visit tragic human sites present higher values in hostility and tourist wellbeing than those who do not. In summary, people who visit more dark places and score higher on negative personality characteristics have higher values of tourist wellbeing.

## 1. Introduction

Many people are increasingly looking for new and unique touristic experiences to satisfy a wide range of motivations. That has driven the segmentation and the emergence of increasingly specific typologies, such as dark tourism, that, in contrast with mass tourism, are characterized by a high degree of diversification and individualization. Dark tourism comprises visiting real or recreated places related with death, suffering, disgrace, or the macabre [1,2]. From the perspective of dark tourism places, it is important to understand what drives people to visit them to design satisfying experiences. We may think of death as an obvious motivation, often part of the site’s history, but it is not always the primary or explicitly recognized motivation for a visit. Sharpley and Stone [3] admitted that the field of motivation to visit dark tourism destinations remains an understudied area, although recent literature has provided an increasing number of empirical studies about the reasons for visiting those sites [4,5].

This research intends to contribute to the dark tourism literature by seeking to understand whether people know what dark tourism is and identify a differentiated sociodemographic, motivational, and tourist practice profile between people who know and do not know what dark tourism is. In addition, it aims to understand if dark tourists’ motivations for visiting dark tourism destinations explain their practices. The research approach relies on empirically exploring the motivations, practices, and sociodemographic characteristics of a sample of 933 people that participated in a survey held in Portugal.

The remainder of the text is organized as follows: firstly, a brief theoretical background is put forward, focused on the dark tourism concept and dark tourists’ motivations and practices; then, the quantitative study’s applied methods and obtained results are described; finally, the results are discussed, and conclusions and implications are drawn.

## 2. Theoretical Background

Despite the fact that some authors consider it one of the older forms of tourism, it has gained great popularity amongst academics from the 1990s onwards [3], confirmed by the significant volume of literature published ever since [4,5,6]. However, understanding the demand for this type of tourism persists as poorly defined and theoretically fragile [3,4,7,8]. For a long time, places that have been the scene of wars, disasters, deaths, and atrocities have always fascinated people, motivating them to travel [3,9]. Sharpley and Stone [3] often use the term dark tourism as the type of tourism that encompasses traveling to sites related to death, suffering, and macabre—a globally accepted definition. However, Tarlow [10] implies the phenomenon is complex by describing it as “visits to places where noteworthy historical tragedies or deaths have occurred that continue to impact our lives”, which raises the question about the inherent motives to consume dark tourism.

### 2.1. Dark Tourists and Their Motivation to Dark Tourism Consumption

Stone’s (2006) idea of dark tourism goes far beyond related attractions. From this standpoint, diverse well-visited tourist sites may become places of dark tourism due to their history linked with death—e.g., suicides in the Eiffel Tower, tombs in the pyramids of Egypt, the Valley of the Kings, and the Taj Mahal, funeral art at the Cairo Museum, and terrorist attacks in Ground Zero [11]. Ashworth and Isaac [12] also suggest that all tourist places have a greater or lesser potential of being perceived as “dark.” Accordingly, the same dark tourism place can evoke different experiences in different visitors (i.e., a site one visitor sees as “dark” may not be for another); thus, the authors argue that no site is intrinsically, automatically, and universally “dark,” as, even they may be labeled as dark, they are not always perceived as such by all visitors.

Walter [13] states that most dark tourism is not specifically motivated, comprising only parallel visits inserted in a trip of a wider reach. Nonetheless, the literature indicates that tourists who visit dark places are not a homogeneous group, and neither the factors inherent to the visitation are the same. Moreover, the “darker” motivation can undertake distinctive levels of intensity. Consequently, in addition to the fascination and interest in death [12,14,15], the visit to this type of place is also motivated by personal, cultural, and psychological reasons [4] or driven by entertainment purposes [7,16].

The literature indicates numerous reasons to visit dark tourism sites: educational experience, desire to learn and understand past events, and historical interest [7,17,18,19,20], as self-discovery purposes [17], identity [7], memory, remembrance, celebration, nostalgia, empathy, contemplation, and homage [10,17,20], curiosity [17,19,20,21], the search for novelty, authenticity, and adventure [2,20], convenience when visiting other places [19], and also status, prestige, affirmation, and recognition that these visits provide [22]. To a lesser extent, the literature also mentions religious and pilgrimage reasons, feelings of guilt, a search for social responsibility, or heritage experience.

The desire to learn and understand stands out as a motive associated with sites of death and/or heritage. Whereas some visitors exhibit a considerable need for emotional experience and connection to their heritage, engaging, as Slade puts it [23], in a “profound heritage experience”, and emotionally to the “dark” space influence [24], other visitors may be knowledge-seekers, who are more interested in a knowledge-enriching experience [25] than an emotional one and look for gaining a deeper understanding. Isaac et al. [20] found that memory, gaining knowledge and awareness, and exclusivity were important motivations for dark tourists; also, “(…), consuming dark tourism may allow the individual a sense of meaning and understanding of past disaster and macabre events that have perturbed life projects” [2]. Tourists’ interest in places associated with death and tragedy may also be related to educational goals [9].

Curiosity and the need to learn and understand are entwined. Dark tourism develops curiosity and satisfies the desire for knowledge of past suffering and pain [26]. Ashworth (2004) and Ashworth and Hartmann [27] suggested three main reasons for visiting dark sites: curiosity about the unusual, attraction to horror, and a desire for empathy or identification with the victims of atrocity. Yan, Zhang, Zhang, Lu and Guo [24] refer to the curious type of dark tourist who engages cognitively by learning about the issue. From another perspective, dark tourists may feel motivated by morbid tourism [28] and show interest in specific macabre exhibitions and museums [29] and fascination with evil [30], given the morbid nature of dark tourism [31]. Other authors present yet other motives: secular pilgrimage; a desire for inner purification; schadenfreude or malicious joy; “ghoulish titillation”; a search for the otherness of death; an interest in personal genealogy and family history; a search for “authentic” places in a commodified world; and a desire to encounter the pure/impure sacred [18]. Iliev [4] concludes that although tourists visit places related to death, they may not necessarily be considered dark tourists; as already acknowledged, those sites may not be experienced as “dark” by each visitor. It is, therefore, imperative that the so-called dark tourists are considered as such based on their experience.

### 2.2. Dark Tourist Personality

Some authors who study dark tourism have tried to relate dark tourist practice with personality characteristics, namely with the dark triad—psychoticism, narcissism, and Machiavellianism [16,32,33,34]. However, the nature of dark tourism, especially that related to the Holocaust, can be so complex that the personality characteristics that motivate it may be less central, so we decided to study the following characteristics: rumination in sadness, self-hatred, hostility, and psychological vulnerability.

Rumination about sadness includes “repetitive thoughts concerning one’s present distress and the circumstances surrounding the sadness” [35]. These thoughts are related to the nature of one’s negative affect, are not goal-directed nor lead to plans for solutional action [36], and are not socially shared while the rumination occurs. Thus, rumination on sadness presents a negative content, “does not facilitate problem resolution, is a solitary activity, and is intrusive if the person is pursuing either self-or situationally imposed task-oriented goals” [35].

Nolen-Hoeksema and Morrow’s [36] measure of rumination focuses on ideation, contrary to expression or disclosure, but it also includes disclosing feelings to others and emotional expressiveness as components of rumination. According to Nolen-Hoeksema and Morrow [36], ruminative responses are different from structured problem-solving because people only think or talk about how “unmotivated, sad, and lethargic they feel” (p. 569). Despite that, Nolen-Hoeksema and Morrow’s [36] stated that ruminative responses include telling others how badly one feels. Although rumination has negative consequences, disclosure may have positive effects [37]; also, some forms of emotional expressiveness, a component of disclosure, seem beneficial [38].

Self-hatred is an “enduring dysfunctional and destructive self-evaluation, characterized by attributions of undesirable and defective qualities, and failure to meet perceived standards and values leading to feelings of inadequacy, incompetency, and worthlessness” [39]. High self-hatred is related to low self-esteem, shame, self-blame or guilt, and a mental state of agitation, raising an experience of psychological and emotional turmoil [39].

According to Derogatis and Melisaratos [40], hostility captures thoughts, feelings, and actions associated with hostile behavior. Although the hostility scale measures perceived levels of expressed hostility rather than actual levels of outwardly expressed hostility, the hostility scale is significantly associated with anger [41], and high anger is related to outward, uncontrolled, and negative expressions of anger [42].

Psychological vulnerability is the “individual’s capacity to deal with mechanisms of maintaining emotional strength, in case of a pessimistic point of view, due to the lack of social support” [43]. Psychological vulnerability is a pattern of cognitive beliefs translating to “a dependence on achievement or external sources of affirmation for one’s sense of self-worth” [44]. Psychological vulnerability is negatively associated with positive affect, self-efficacy, and social support and positively associated with negative affect, perceived powerlessness, and maladaptive coping behavior [43,44]. Dark tourists are subjects situated in emotionally sensitive spaces [45] that can trigger their psychological vulnerability.

### 2.3. Research Questions

Although research on dark tourism has increased in recent years, there are not enough studies exploring if people’s knowledge of this phenomenon and their personality traits lead to distinctive dark tourists’ motivations and behaviors. Taking into account the aforementioned motivations to visit dark tourism places, the present study intends to empirically explore if dark tourists’ personality characteristics and sociodemographic variables impact such motivations and dark tourists’ practices and wellbeing (the latter, measured as a dark tourism practice index, given the diversity of known dark tourism practices). Specifically, our research questions are: Do rumination on sadness, self-hatred, hostility, and psychological vulnerability explain the practices and motivations for dark tourism and thus explain tourist wellbeing? Does knowing what dark tourism is (or not) impact rumination on sadness, self-hatred, hostility, and psychological vulnerability, as well as practices and motivations for dark tourism and tourist wellbeing?

## 3. Materials and Methods

Given the research questions, the aims of the present study are as follows: (1) to find the sociodemographic differences in touristic practices and motivations for dark tourism according to two groups (those who knew what dark tourism is and those who did not know); (2) to assess the fit of the rumination on the sadness scale, self-hatred scale, hostility scale, psychological vulnerability scale, and tourism wellbeing scale; (3) to determine the differences in rumination on sadness, self-hatred, hostility, psychological vulnerability, and tourism wellbeing according to two groups (those who knew what dark tourism is and those who didn’t know); (4) to find the differences in rumination on sadness, self-hatred, hostility, psychological vulnerability, and tourism wellbeing according to practices and motivations for dark tourism; and (5) to determine variables that contribute to the dark tourism practice index. Accordingly, we hypothesize:

**H1.**  *Participants who know what dark tourism is are younger and have more education than those who do not.*

**H2.**  *Participants who know what dark tourism is are more motivated and visit more places associated with dark tourism than those who do not.*

**H3.**  *All measures show a good fit for the sample.*

**H4.**  *Differences in rumination on sadness, self-hatred, hostility, psychological vulnerability, and tourism wellbeing according to two groups (those who knew what dark tourism is and those who did not know) will be found.*

**H5.**  *Differences in rumination on sadness, self-hatred, hostility, psychological vulnerability, and tourism wellbeing according to practices and motivations for dark tourism will be found.*

**H6.**  *Gender, age, to know/know not dark tourism, and the motivations of curiosity, need to learn, need to understand, and pleasure will contribute to explaining dark tourism practice.*

### 3.1. Procedures

All procedures followed the Declaration of Helsinki and later amendments or comparable ethical standards. The investigation protocol included informed consent, and confidentiality and anonymity of the data were guaranteed. The research protocol was applied in person to a random sample of participants between 18 October and 17 December 2021. The participants were informed about the study’s purpose and were ensured confidentiality and anonymity of the data; they also signed informed consent. The inclusion criteria consisted of being over 18 years old, Portuguese, and having touristic experiences. The respondents were approached by two researchers and five MSc students on the University’s campuses and within their informal networks, with the questionnaire being self-administered.

### 3.2. Instruments

The instruments that were not validated for the Portuguese population—the Rumination on Sadness Scale (RSS) and the Self-Hatred Scale (SHS)—were first translated from English to Portuguese by two bilingual translators, one from and another not from the field of psychology. Then, a third bilingual translator from the field of psychology provided a reconciliation of the two translations. Next, a native English speaker not from the psychology field independently performed the reconciled version’s back-translation. Finally, the first translator reviewed the back-translated version of the scale and compared it with the original English version to ensure linguistic and cultural equivalence consistency.

- *Sociodemographic questionnaire*

The sociodemographic questionnaire included questions related to gender (feminine—0; masculine—1), age, education (no education–0; primary education—1; secondary education—2; higher education—3), marital status (no relationship-single, divorced, separated, widowed–0; in a relationship-boyfriends, married, de facto union—1), and employment status (inactive—unemployed, retired, on sick leave–0; active-student, employee, housewife, caregivers—1).

- *Questionnaire about dark tourism’s practices*

The questionnaire on dark tourism practices includes a question about knowledge of dark tourism (or not). In addition, it also asked participants about their tourist practices related to dark tourism (Have you ever visited…? cemeteries; holocaust museums; sites of human tragedy; concentration camps; prisons; sites of war; sites of natural disasters; stop to see accidents). All these questions are answered dichotomously (no—0; yes—1).

- *Questionnaire about dark tourism´s motivations*

This questionnaire includes the presentation of several reasons to visit a dark place: curiosity, the need to learn, the need to see, the need to understand, pleasure, and the need to see morbid things. All these questions are answered dichotomously (no—0; yes—1).

- *Rumination on Sadness Scale (RSS)*

The Rumination on Sadness Scale, an individual-difference measure of rumination on sadness, was developed by Conway et al. [35] as an alternative to the Ruminative Responses Scale of the Response Styles Questionnaire (RRRSQ; [36]). It is a unifactorial scale with 13 items. Higher ratings indicate higher levels of rumination on sadness. Cronbach’s alpha, the internal reliability coefficient, was 0.91 in the original version. Since there is no Portuguese version of this scale, it will be validated in this study.

- *Self-Hatred Scale (SHS)*

The Self-Hatred Scale was developed by Turnell et al. [39] to assess individuals’ levels of self-hatred. Since self-hatred is a significant predictor of suicidal ideation, this scale has the potential to be helpful in suicide risk assessment. Higher ratings indicate higher levels of self-hatred. Cronbach’s alpha was 0.95 in the original version. There is no Portuguese version of this scale, so it will also be validated in this study.

- *BSI Hostility Scale (HSS)*

BSI Hostility Scale (HS) is a subscale of the Brief Symptoms Inventory [BSI; [40]], whose Portuguese version is from Canavarro [46]. BSI is a 53-item measure to identify self-reported clinically relevant psychological symptoms in adolescents and adults. The BSI covers nine symptom dimensions: Somatization, Obsession-Compulsion, Interpersonal Sensitivity, Depression, Anxiety, Hostility, Phobic Anxiety, Paranoid Ideation, and Psychoticism; and three global indices of distress: Global Severity Index, Positive Symptom Distress Index, and Positive Symptom Total. The Hostility subscale includes five items, and higher ratings indicate higher levels of hostility. In the original version, the alpha coefficients for the nine dimensions of the scale ranged from 0.64 in the Psychoticism dimension to 0.81 in the Somatization dimension. In the Portuguese version, the alpha coefficients ranged from 0.71 in the Psychoticism dimension to 0.85 in the Depression dimension.

- *Psychological Vulnerability Scale (PVS)*

The Psychological Vulnerability Scale (PVS) was designed to obtain information about maladaptive cognitive patterns, such as dependence, perfectionism, need for external sources of approval, and generalized negative attributions. The PVS is a six-item scale with higher scores indicating greater psychological vulnerability. In the original version [44], Cronbach’s α coefficient ranged from 0.71 to 0.87 for different samples; in the Portuguese version [47], Cronbach’s α coefficient was 0.73.

- *Tourism Wellbeing Scale (TWS)*

The Tourism Wellbeing Scale (TWS) was developed by [48] Garcês et al. (2018 [49]); it aims to evaluate tourism wellbeing in each destination, having been built from positive psychology variables, namely, wellbeing, creativity, optimism, and spirituality. It is a unifactorial scale with eight items. Higher ratings indicate higher levels of tourism wellbeing. Cronbach’s alpha was 0.97 in the original version.

### 3.3. Data Analysis

Prior to analysis, the normality of items was examined by skewness (SI) and kurtosis (KI) indexes; absolute values of SI less than 3 and KI less than 10 indicate a normal distribution of the data. [50]. All the instruments were subject to a confirmatory factor analysis (CFA) procedure with maximum likelihood estimation (MLE). The model fit evaluation was based on test statistics and approximate fit indexes, following the thresholds presented in Kline [50]. Thus, a non-significant model chi-square statistic, χ^2^, states that the model fits the data acceptably in the population; the higher the probability related to χ^2^, the closer the fit to the perfect fit. A value of the parsimony-corrected index Steiger–Lind root mean square error of approximation (RMSEA) close to 0 represents a good fit; RMSEA ≤ 0.05 may indicate a good fit, but the upper bound of the 90% confidence interval exceeding 0.10 may indicate poor fit; also, this test should be non-significant at the 0.05 level. Values of incremental fit index (IFI), Tucker–Lewis index (TLI), and the Bentler incremental comparative fit index (CFI), close to 1 (0.95 or better), are indicators of best fit; also, the standardized root mean square residual (SRMR), a statistic related to the correlation residuals (SRMR over 0.10 suggests fit problems) was used; the smallest the values, the most parsimonious is the model.

Besides goodness-of-fit index evaluation, model re-specification involved analyzing path estimates, standardized residuals of items, and modification indices for all non-estimated parameters. The modifications indices (MI) provide information about potential cross-loadings and error term correlations not specified in the model and the expected change in the chi-square value for each fixed parameter if it were to be freed. Only modifications theoretically meaningful and MI > 11 were considered. Finally, Cronbach’s alpha coefficients were calculated to ascertain the model’s reliability.

Group differences were analyzed. The independent t-test was applied to compare the means of the two groups. In addition, chi-squared was used to compare distributions’ differences and Mann–Whitney test to compare ordinal data. Three measures of the effect size, Cohen’s d, the eta squared, phi, and rank biserial correlation were used according to the variables’ measurement level; interpretation followed Cohen’s [51] guidelines; the statistical significance level was set at 0.05. Statistical analysis was performed using SPSS version 28 and AMOS version 28.

## 4. Results

The sample includes 993 participants, mainly female, in a romantic relationship, with secondary or university education, and active; the mean age is around 31 years. Statistically significant differences were found concerning age and education between the sample that had already heard about dark tourism and knew what it was and the sample that had not yet heard about it. Participants who had heard about dark tourism were significantly younger and more educated than those who had not (Table 1).

Concerning the total sample and dark tourism practices, most people have visited cemeteries, and about a third of the sample stopped to see accidents. On the other hand, about a quarter of the sample already had other practices, except for a visit to concentration camps, which was only carried out by about 14% of the total sample. The same trend remains in the sample that has not yet heard about dark tourism and the sample that has. However, there are statistically significant differences between these two samples regarding practices related to dark tourism, being that the sample that has already heard about dark tourism visits many more Holocaust museums, sites of human tragedy, concentration camps, prisons, and sites of natural disasters than the sample that has not yet heard about dark tourism (Table 2).

As for the reasons behind the desire to visit dark places, curiosity stands out in the total sample, with the least chosen reason being the need to see morbid things. The same trend can be seen in the two subsamples. However, there are statistically significant differences between these two samples regarding motives to visit dark places, being that the sample that has already heard about dark tourism presents higher values in the motives related to curiosity, the need to learn and understand, and the need to see morbid things than the sample that has not yet heard about dark tourism (Table 3).

Table 4 shows the descriptive statistics related to the items of the instruments used in this study: the rumination on sadness, tourism wellbeing, self-hatred, hostility, and psychological vulnerability. The skewness and kurtosis values are all within the normative values, ensuring the normality of the distribution, except for item SHS3 whose values are slightly above the recommended one.

**Figure 1:**
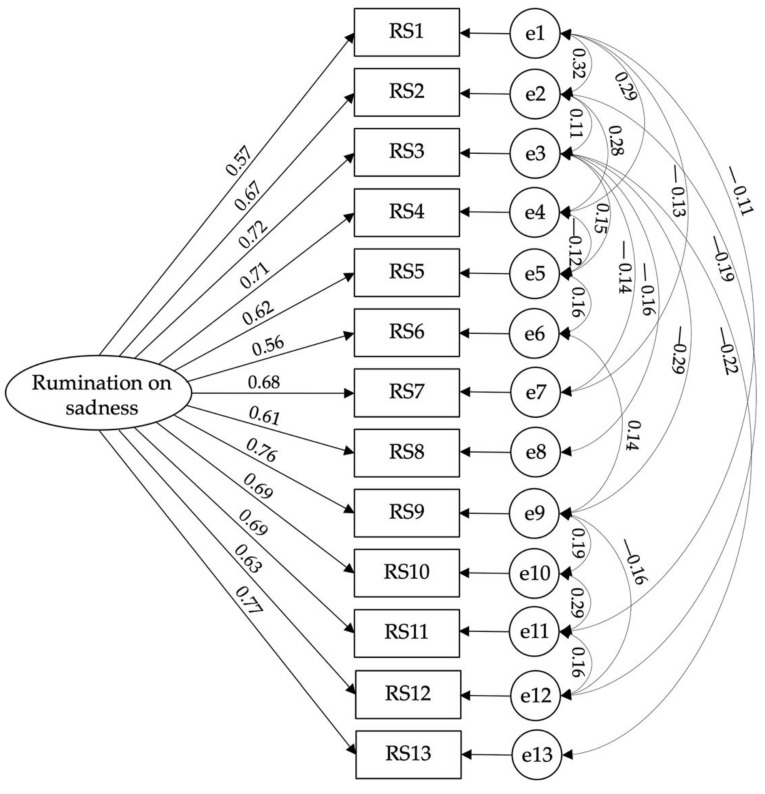
Model fit of Rumination on Sadness Scale.

A confirmatory factorial analysis of the rumination on sadness scale was carried out to confirm the authors’ model [χ^2^(46) = 4.121; CFI = 0.977; TLI = 0.961; IFI = 0.977; RMSEA = 0.056; PCLOSE = 0.107: SMRM = 0.028]; however, to achieve this model fit, some correlations between errors were established (Figure 1).

**Figure 2:**
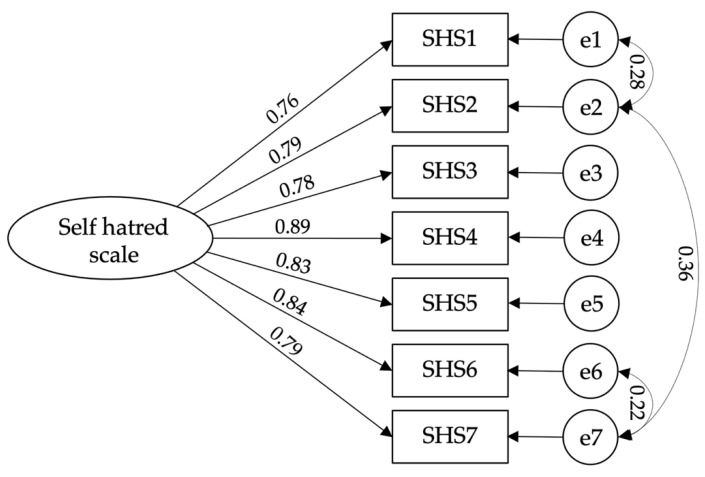
Model fit of Self-hatred Scale.

**Figure 3:**
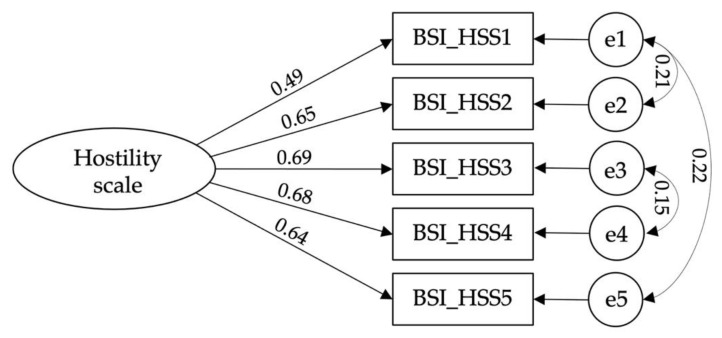
Model fit of Hostility Scale.

**Figure 4:**
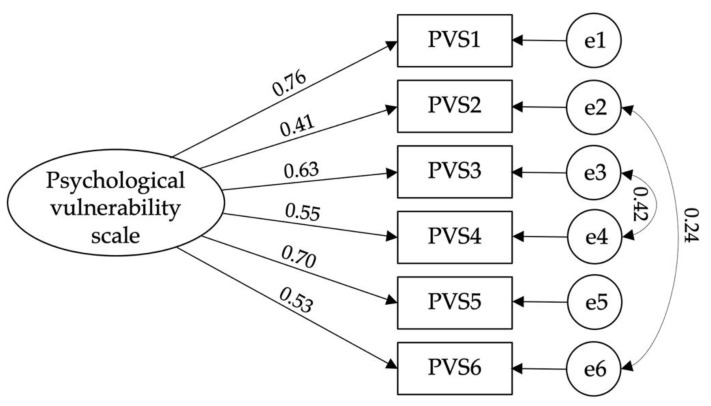
Model fit of Psychological Vulnerability Scale.

**Figure 5:**
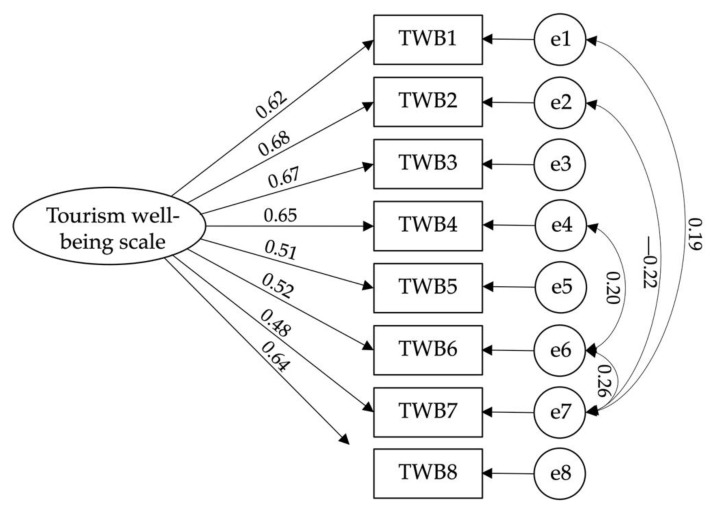
Model fit of Tourism Wellbeing Scale.

Confirmatory factorial analysis of the self-hatred scale [χ^2^(11) = 5.118; CFI = 0.992; TLI = 0.984; IFI = 0.992; RMSEA = 0.064; PCLOSE = 0.069: SMRM = 0.015] (Figure 2), hostility scale [χ^2^(2) = 4.216; CFI = 0.995; TLI = 0.976; IFI = 0.995; RMSEA = 0.057; PCLOSE = 0.317: SMRM = 0.012] (Figure 3), psychological vulnerability scale [χ^2^(7) = 2.886; CFI = 0.992; TLI = 0.983; IFI = 0.992; RMSEA = 0.044; PCLOSE = 0.644; SMRM = 0.018] (Figure 4), and tourism wellbeing scale [χ^2^(16) = 3.787; CFI = 0.979; TLI = 0.964; IFI = 0.980; RMSEA = 0.053; PCLOSE = 0.339: SMRM = 0.029] (Figure 5) were carried out to assess the models’ adjustments. Despite finding good fits for all models, some correlations between errors were established to achieve such fits. Thus, hypothesis H3 is confirmed.

There are no differences in the values of rumination on sadness, self-hatred, hostility, psychological vulnerability, and tourism wellbeing concerning knowing what dark tourism is or not (Table 5).

Differences were assessed regarding the values of rumination on sadness, self-hatred, hostility, psychological vulnerability, and tourism wellbeing according to dark tourism practices. Being that only statistically significant results are presented, it was found that participants who visit cemeteries have significantly lower values of self-hatred and psychological vulnerability than participants who report not visiting cemeteries (Table 6). Furthermore, those who visit tragic human sites present higher values in hostility and tourism wellbeing than those who do not. Those who visit sites of war present higher values in self-hatred than those who did not. Those who visit site of natural tragedies also present higher values in hostility and tourism wellbeing. Lastly, those who stop to see accidents present higher values in rumination on sadness, self-hatred, hostility, psychological vulnerability, and tourism wellbeing than those who do not stop (Table 6).

Differences were also assessed concerning the values of rumination on sadness, self-hatred, hostility, psychological vulnerability, and tourism wellbeing according to dark tourism motives. Those participants who identified curiosity, need to see, and need to understand as reasons to visit dark places in the context of tourism presented higher values in rumination on sadness, self-hatred, hostility, psychological vulnerability, and tourism wellbeing than those who did not identify curiosity as a motive (Table 7). Concerning the motive “need to learn”, it was found to be a statistically significant difference in tourism wellbeing, being that those who identified the need to learn as a motive to visit dark places in the context of tourism present higher values in tourism wellbeing and self-hatred than those who did not. Those participants who identified the need to see as a reason to visit dark places in the context of tourism presented higher values in rumination on sadness, self-hatred, hostility, and psychological vulnerability than those who did not identify the need to see as a motive (Table 7). Those participants who recognized the need to understand as a reason to visit dark places in the context of tourism present higher values in rumination on sadness, hostility, psychological vulnerability, and tourism wellbeing than those who did not identify the need to understand as a motive (Table 7). Concerning the motive “pleasure”, it was found a statistically significant difference in tourism wellbeing; those who recognized pleasure as a motive to visit dark places presented higher values in tourism wellbeing than those who did not. Lastly, those participants who identified the need to see morbid things as a reason to visit dark places presented higher values in rumination on sadness, self-hatred, hostility, and psychological vulnerability than those who did not identify the need to see morbid things as a motive (Table 7).

After creating a new variable, an index about practices related to dark tourism, based on the individual items, we carried out a multiple linear regression in which the dependent variable is the index, and the independent variables are the motivations, with the intent to find the variables that explain the touristic practice. It was found that gender, age, know/know not dark tourism, and motives (curiosity, need to learn, need to understand, and pleasure) explain 38% of the touristic practice (Table 8).

## 5. Discussion

The aims of the present study were to find the sociodemographic differences in touristic practices and motivations for dark tourism according to two groups (those who knew what dark tourism is and those who did not know); to determine the differences in rumination on sadness, self-hatred, hostility, psychological vulnerability, and tourism wellbeing according to two groups; to find the differences in rumination on sadness, self-hatred, hostility, psychological vulnerability, and tourism wellbeing according to practices and motivations for dark tourism; and, at last, to determine variables that contribute to a dark tourism practice index. To this end, we carried out a cross-sectional study that included questionnaires related to sociodemographic aspects, motivations to visit dark tourism places, practices of dark tourism, the rumination on the sadness scale, the self-hatred scale, the hostility scale, the psychological vulnerability scale, and the tourism wellbeing scale.

Concerning the participants’ profiles, those who had heard about dark tourism were significantly younger and more educated than those who had not. These results confirm hypothesis H1. These results corroborate those of Millán, et al. [52] who found a profile of dark tourists in Cordoba between 26 and 40 years old and having university studies. Dark tourism is a niche market [53] and also is itself a trend [54], and young people are more available and attentive to new trends [55]. In addition, more educated people seek more information and have superior technological skills [56]. Significant differences between the two samples regarding practices related to dark tourism were found, being that the sample that has already heard about dark tourism visits much more Holocaust museums, sites of human tragedy, concentration camps, prisons, and sites of natural disasters than the sample that has not yet heard about dark tourism. These results confirm hypothesis H2. According to Iliev [4], “if tourists do not experience a site as dark, then they cannot be called dark tourists”, so the author proposed a more apparent distinction of the “dark tourists” based on experience. Ashworth and Isaac (2015) also stated that any tourist site has a greater or lesser potential of being perceived as “dark.” Besides, “darkness cannot be viewed as an objective fact because it is subjectively and socially constructed since (different) people in various (cultural or social) contexts understand and experience dark tourism in different ways” [57]. In fact, we may ask “who makes the association of ‘darkness’ to a place? Is the label ‘dark tourism’ applied by those offering (and commoditizing) the visitor experience? Alternatively, is any “dark” significance to be evaluated and decided upon by the tourists themselves?” [58]. “Dark tourism consumption can no longer be derived as an ordinary activity where humans might engage in for “fun”, but rather as part of a quest for a deeper experience, especially in our inherent fear of death” [4].

The subsample that has already heard about dark tourism presents higher values in the curiosity, the need to learn and understand, and the need to see morbid things motives than the sample that has not yet heard about dark tourism. These results also confirm hypothesis H2. In fact, dark tourists are very interested in understanding historical events; they are psychologically moved by the need to be in contact with authentic experiences by looking at the other’s death as if it were their own death [59]. One of the motivations that drive dark tourists is the possibility of re-creating the same emotions victims experienced, followed by the authenticity issue [60]. “Many dark tourists are motivated by the desire and interest in cultural heritage, learning, education, understanding about what happened at the dark site” [4].

There are no differences in the values of rumination on sadness, self-hatred, hostility, psychological vulnerability, and tourism wellbeing concerning knowing what dark tourism is or not. Therefore, hypothesis H4 cannot be confirmed. These results apparently seem to contradict the relationship between the dark triad of the personality (narcissism, Machiavellianism, and psychopathy) and the practice of dark tourism [16,32,33,34]. That relationship, studied by those authors, reflects the practice of dark tourism and not the knowledge about it (which is the subject of our study), although there is hardly any knowledge without practice. Concerning tourism wellbeing, these results may question Kidron [61] who said that dark tourism generates wellbeing and thus assume that dark tourists show wellbeing despite dark practices. However, our results do not show greater wellbeing in the participants who knew in advance what dark tourism was in relation to the others.

Participants who visit cemeteries have significantly lower values of self-hatred and psychological vulnerability than participants who report not visiting cemeteries. Visiting a cemetery can fulfill different functions, such as visiting a dark place or the social and cultural function of honoring the dead. Probably, our results reflect this last function to the detriment of the first and this conformity to cultural and social practices is in accordance with lower values of psychopathology [62], namely rumination on sadness, self-hatred, hostility, and psychological vulnerability. This result partially confirms hypothesis 5.

Those who visit sites of war present higher levels of self-hatred than those who did not. Furthermore, those who visit natural tragedies sites present higher values in hostility and tourism wellbeing than those who do not. This result reflects the relationship of this tourist practice with the above-mentioned dark triad [16,32,33,34] and is in line with Kidron [61], who suggested wellbeing in dark tourists. At last, those who stop to see accidents present higher values in rumination on sadness, self-hatred, hostility, psychological vulnerability, and tourism wellbeing than those who do not stop. Again, this result reveals the relationship between psychopathology and tourist wellbeing that needs to be further explained, although some authors suggest that psychopathology leads to less tourism wellbeing [63]. This result partially confirms hypothesis 5.

Participants who identified curiosity as a reason to visit dark places in the context of tourism presented higher values in rumination on sadness, self-hatred, hostility, psychological vulnerability, and tourism wellbeing than those who did not identify curiosity as a motive. Curiosity has been a central reason pointed out in the literature for tourism in general [64] and, specifically, for dark tourism [15,17,19,20,21,65,66]. Curiosity is a complex construct, which can be seen as something positive, but it can also contain darker aspects of the personality, namely morbid curiosity, and this fact explains its relationship with, on the one hand, wellbeing, and, on the other hand, with rumination on sadness, self-hatred, hostility, and psychological vulnerability. This result partially confirms hypothesis 5.

The participants who identified the need to learn, the need to understand as motives to visit dark places in the context of tourism present higher values in tourism wellbeing and self-hatred than those who did not. The need to learn and understand are also central reasons for tourism in general and their relationship with wellbeing does not seem specific to dark tourism [67]. This result partially confirms hypothesis 5.

The participants who identified the need to see as a reason to visit dark places in the context of tourism presented higher values in rumination on sadness, self-hatred, hostility, psychological vulnerability, and tourism wellbeing. This result partially confirms hypothesis 5. Similarly to the need to learn, the need to see correlates with wellbeing but with psychopathology. Perhaps this need to learn motivation is correlated with the touristic practice of seeing morbid things [68].

The participants who recognized pleasure as a motive to visit dark places presented higher values in tourism wellbeing than those who did not. This result partially confirms hypothesis 5. Dark tourism conforms with the pleasure of tourism in general (Yanjun et al., 2015); wellbeing derives from the emotional experience of dark tourism as a motor for transforming the self [69].

The participants who identified the need to see morbid things as a drive to visit dark places presented higher values in rumination on sadness, self-hatred, hostility, and psychological vulnerability. The need to see morbid things may be a specific motivation for dark tourism [1,70] and not tourism in general. To that extent, the relationship between this motivation and rumination on sadness, self-hatred, hostility, and psychological vulnerability is justified. This result partially confirms Hypothesis 5.

The reasons to visit dark places-curiosity, the need to see, the need to understand, and pleasure are positively and significantly correlated with all places associated with dark tourism. Gender, age, know/know not dark tourism, and motives (curiosity, the need to learn, the need to understand, and pleasure) explained 38.1% of the practice index variance, thus confirming H6. These results mean that motivations to visit dark places are associated with the touristic activity itself and may contradict those of Buda [71], that claims more emotional and psychoanalytical explorations through the concepts of the death drive [71], desire [72], and unconsciousness and voyeurism [73]. In fact, dark tourists are not altruistic persons [14,60]. Moreover, Jovanovic, Mijatov, and Šuligoj [32] found that Machiavellianism was related to the preference for dark exhibitions, psychopathy to the preference for visiting conflict/battle sites, and sadism was negatively related to the preference for fun factories and dark tourism sites. However, the “darker” motivation may present different levels of intensity; besides the fascination and interest in death [15], these visits are also motivated by personal, cultural, and psychological reasons [4] and/or by entertainment purposes such as entertainment-based museums of torture [7,16]. One of the most curious outcomes of this study is the association of motivations to visit dark tourist sites and self-hatred; the fact that the authors have not found any study that could explain such a result suggests this association exists in the context of dark tourism and not of tourism in general. The dark nature of this type of tourism can be attractive to tourists with less positive personality traits such as self-hatred.

## 6. Conclusions

The results of this study add new knowledge to this area of expertise as it allows us to understand the association between motivations and practices related to dark tourism. This study also identified the main motivations to visit dark places-curiosity, the need to see, the need to understand, and pleasure, being, interestingly, all internal motivations and, thus, contradicting the literature that, in addition to these motivations, also identifies external motivations. Most findings also indicate that the rumination on sadness, self-hatred, hostility, and psychological vulnerability personality dimensions are associated with dark practices (e.g., the need to see morbid things). Lastly, people who visit more dark places and score higher on negative personality characteristics have higher values of tourism wellbeing. These findings are in line with the literature, which suggests that dark tourism generates negative and positive wellbeing (or even ambivalence). As such, dark tourists, even presenting negative personality characteristics, and also because of them, show tourism wellbeing in their practices and motivations.

The fact that this study was held in a specific sample in Portugal may be considered a limitation; future lines of research could extend it to other countries and age segments.

## Figures and Tables

**Table 1:** Sample sociodemographic characteristics.

Sociodemographic Variables		*N* (%)	*n* (%)	*n* (%)	χ^2^	*p*	Φ
		Total	Know Not Dark Tourism	Know Dark Tourism			
Sample		993 (100.0)	600 (60.4)	393 (39.6)			
Gender	Female	574 (57.8)	343 (57.2)	231 (68.8)	0.253	0.615	−0.016
	Male	419 (42.2)	257 (42.8)	162 (41.2)			
Marital status	No relation	377 (38.0)	224 (37.3)	153 (38.9)	0.257	0.612	−0.016
	In a relation	616 (62.0)	376 (62.7)	240 (61.1)			
Education	No education	9 (0.9)	6 (1.0)	3 (0.8)	18.955	**<0.001**	0.139
	Primary	77 (7.8)	63 (10.5)	14 (3,6)			
	Secondary	437 (44.0)	268 (44.7)	169 (43.0)			
	University	470 (47.3)	263 (43.8)	207 (52.7)			
Professional status	Inactive	110 (11.1)	72 (12.0)	38 (9.7)	1.310	0.252	0.036
	Active	883 (88.9)	528 (88.0)	355 (90.3)			
					***t***	***p***	***d***
Age	M ± SD; Min–Max	31.28 ± 14.45; 18–87	32.41 ± 15.07; 18–87	29.56 ± 13.29; 18–79	3.136	**0.002**	0.198

Notes: *N* = frequencies; % = percentage; *M* = mean; *SD* = standard deviation; χ^2^ = qui-squared test; Φ = Phi size effect; *t* = *t*-test; Cohen’s *d* = size effect; *p* = *p*-value. In bold: statistically significant values.

**Table 2:** Dark tourism practices.

		Total		Know Not Dark Tourism		Know Dark Tourism				
Sample		*N*	%	*n*	%	*n*	%	χ^2^	*p*	Φ
Have you ever visited…?		993	100	600	60.4	393	39.6			
Cemeteries	No	291	29.3	178	29.7	113	28.8	0.096	0.057	0.010
	Yes	702	70.7	422	70.3	280	71.2			
Holocaust Museums	No	762	76.7	480	80.0	282	71.8	9.041	**0.003**	0.095
	Yes	231	23.3	120	20.0	111	28.2			
Sites of Human Tragedy	No	768	77.3	490	81.7	278	70.7	16.184	**<0.001**	0.128
	Yes	225	22.7	110	18.3	115	29.3			
Concentration Camps	No	855	86.1	531	88.5	324	82.4	7.281	**0.007**	0.086
	Yes	138	13.9	69	11.5	69	17.6			
Prisons	No	748	75.3	479	79.8	269	68.4	16.563	**<0.001**	0.129
	Yes	245	24.7	121	20.2	124	31.6			
Sites of War	No	783	78.9	485	80.8	298	75.8	3.569	0.059	0.060
	Yes	210	21.1	115	19.2	95	24.2			
Sites of Natural Disasters	No	769	77.4	485	80.8	284	72.3	9.980	**0.002**	0.100
	Yes	224	22.6	115	19.2	109	27.7			
Stop to see accidents	No	622	62.6	380	63.3	242	61.6	0.313	0.576	0.018
	Yes	371	37.4	220	36.7	151	38.4			

Notes: *N* = frequencies; % = percentage; χ^2^ = qui-squared test; Φ = Phi size effect; *p* = *p*-value. In bold: statistically significant values.

**Table 3:** Dark tourism motives.

		Total		Know Not Dark Tourism		Know Dark Tourism				
Sample		*N*	%	*n*	%	*n*	%	χ^2^	*p*	Φ
Motives to visit		993	100	600	60.4	393	39.6			
Curiosity	No	417	42.0	273	45.5	144	36.6	7.605	**0.006**	0.088
	Yes	576	58.0	327	54.5	249	63.4			
Need to learn	No	675	68.0	433	72.2	242	61.6	12.231	**<0.001**	0.111
	Yes	318	32.0	167	27.8	151	38.4			
Need to see	No	636	64.0	392	65.3	244	62.1	1.087	0.297	0.033
	Yes	357	36.0	208	34.7	149	37.9			
Need to understand	No	591	59.5	390	65.0	201	51.1	18.919	**<0.001**	0.138
	Yes	402	40.5	210	35.0	192	48.9			
Pleasure	No	913	91.9	557	92.8	356	90.6	1.620	0.203	0.040
	Yes	80	8.1	43	7.2	37	9.4			
Need to see morbid things	No	941	94.8	576	96.0	365	92.9	4.672	**0.031**	0.069
	Yes	52	5.2	24	4.0	28	7.1			

Notes: *N* = frequencies; % = percentage; χ^2^ = qui-squared test; Φ = Phi size effect; *p* = *p*-value. In bold: statistically significant values.

**Table 4:** Items’ frequencies.

	Minimum	Maximum	Mean	Standard Deviation	Skewness	Kurtosis
Rumination on sadness (RSS)						
RSS1	1	5	2.31	1.18	0.62	−0.46
RSS2	1	5	2.68	1.28	0.30	−0.98
RSS3	1	5	2.62	1.30	0.32	−0.99
RSS4	1	5	2.26	1.20	0.69	−0.52
RSS5	1	5	2.11	1.28	0.92	−0.34
RSS6	1	5	1.85	1.16	1.28	0.68
RSS7	1	5	2.25	1.30	0.70	−0.69
RSS8	1	5	1.54	0.96	1.94	3.31
RSS9	1	5	1.93	1.17	1.13	0.29
RSS10	1	5	2.21	1.17	0.72	−0.38
RSS11	1	5	2.19	1.23	0.68	−0.65
RSS12	1	5	2.94	1.28	0.02	−1.06
RSS13	1	5	2.00	1.25	1.06	−0.04
Self-hatred (SHS)						
SHS1	1	7	1.52	1.12	2.37	5.14
SHS2	1	7	1.73	1.28	1.95	3.23
SHS3	1	7	1.31	0.88	3.59	13.83
SHS4	1	7	1.65	1.24	2.10	3.76
SHS5	1	7	1.52	1.14	2.56	6.40
SHS6	1	7	1.79	1.50	2.05	3.29
SHS7	1	7	1.94	1.53	1.73	2.18
Hostility (HSS)						
BSI_HSS1	0	4	2.33	1.23	−0.17	−1.02
BSI_HSS2	0	4	1.31	1.22	0.70	−0.43
BSI_HSS3	0	4	0.85	1.01	1.32	1.34
BSI_HSS4	0	4	0.98	1.13	1.18	0.70
BSI_HSS5	0	4	1.57	1.27	0.46	−0.85
Psychological vulnerability (PVS)						
PVS1	1	5	2.85	1.37	0.19	−1.18
PVS2	1	5	2.73	1.36	0.27	−1.12
PVS3	1	5	2.02	1.28	1.03	−0.16
PVS4	1	5	2.00	1.19	1.04	0.07
PVS5	1	5	2.53	1.36	0.44	−1.04
PVS6	1	5	3.19	1.44	−0.16	−1.33
Tourism wellbeing (TWB)						
TWBS1	1	7	4.50	1.78	−0.43	−0.69
TWBS2	1	7	5.74	1.54	−1.31	1.06
TWBS3	1	7	4.89	1.57	−0.54	−0.39
TWBS4	1	7	5.41	1.66	−0.97	0.11
TWBS5	1	7	4.30	1.92	−0.28	−1.05
TWBS6	1	7	4.18	2.00	−0.17	−1.13
TWBS7	1	7	3.48	2.03	0.23	−1.21
TWBS8	1	7	5.06	1.76	−0.75	−0.28

**Table 5:** Rumination on sadness (RSS), self-hatred (SHS), hostility (HSS), psychological vulnerability (PVS), and tourism wellbeing (TWBS) frequencies and differences between those who know dark tourism and those who do not.

		Total		Know Not Dark Tourism	Know Dark Tourism			
	α	*M*	*SD*	*MR*	*MR*	*U*	*p*	*r*
Sample		993	100	600	393			
RSS Total	0.916	2.22	0.86	498.37	494.91	117,077.500	0.852	−0.0059
SHS Total	0.931	1.64	1.06	504.99	484.80	113,106.500	0.245	−0.0369
HSS Total	0.790	1.41	0.87	498.43	4.94.82	117,044.500	0.846	−0.0062
PVS Total	0.788	2.55	0.93	508.70	479.13	110,878.500	0.112	−0.0505
TWBS Total	0.818	4.69	1.19	486.39	513.19	111,536.500	0.150	−0.0457

Notes: α = Cronbach’s alpha; *M* = mean; *SD* = standard deviation; MR–mean rank; *U* = Mann–Whitney test; *p* = *p*-value; *r* = rank-biserial correlation.

**Table 6:** Rumination on sadness (RSS), self-hatred (SHS), hostility (HSS), psychological vulnerability (PVS) and tourism wellbeing (TWBS) frequencies and differences according to dark tourism practices.

	No Visit	Visit			
Sample	*MR*	*MR*	*U*	*p*	*r*
**Cemeteries**					
SHS Total	550.65	474.76	86,528.500	**<0.001**	−0.1291
PVS Total	592.94	483.34	92,554.500	**0.020**	−0–0741
**Sites of war**					
SHS Total	485.08	541.46	72,879.000	**0.007**	−0.0861
**Sites of Natural Disasters**					
HSS Total	479.27	557.88	72,491.500	**<0.001**	−0.1148
TWBS Total	483.55	543.17	75,786.000	**0.006**	−0.0869
**Stop to see accidents**					
RSS Total	463.43	553.28	94,502.500	**<0.001**	−0.1516
SHS Total	471.10	540.42	99,273.500	**<0.001**	−0.1253
HSS Total	461.28	556.89	93,161.000	**<0.001**	−0.1605
PVS Total	457.84	562.66	91,021.500	**<0.001**	−0.1771
TWBS Total	475.07	533.77	101,739.000	**0.002**	−0.0991

Notes: α = Cronbach’s alpha; *M* = mean; *SD* = standard deviation; MR–mean rank; *U* = Mann–Whitney test; *p* = *p*-value; *r* = rank-biserial correlation. In bold: statistically significant values.

**Table 7:** Rumination on sadness (RSS), self-hatred (SHS), hostility (HSS), psychological vulnerability (PVS), and tourism wellbeing (TWBS) frequencies and differences according to dark tourism motives.

	No	Yes			
Sample	*MR*	*MR*	*U*	*p*	*r*
**Curiosity**					
RSS Total	466.19	519.31	107,248.000	**0.004**	−0.0915
SHS Total	454.48	527.78	102,364.000	**<0.001**	−0.1353
HSS Total	457.02	525.95	103,423.000	**<0.001**	−0.1188
PVS Total	449.50	531.39	100,288.000	**<0.001**	−0.1411
TWBS Total	468.48	517.65	108,203.000	**0.008**	−0.0847
**Need to learn**					
SHS Total	480.57	531.88	96,232.000	**0.005**	−0.0895
TWBS Total	481.97	528.91	97,177.500	**0.016**	−0.0764
**Need to see**					
RSS Total	468.92	547.02	95,668.500	**<0.001**	−0.1307
SHS Total	479.58	528.04	102,446.000	**0.006**	−0.0869
HSS Total	469.66	545.71	96,137.000	**<0.001**	−0.1276
PVS Total	477.48	431.78	101,110.500	**0.004**	−0.0910
TWBS Total	482.84	522.22	104,523.000	**0.038**	−0.0659
**Need to understand**					
RSS Total	462.18	548.20	98,210.500	**<0.001**	−0.1473
SHS Total	479.19	523.19	108,263.000	**0.011**	−0.0807
HSS Total	476.29	527.45	106,551.500	**0.006**	−0.0878
PVS Total	476.42	527.26	106,625.500	**0.006**	−0.0871
TWBS Total	479.01	523.45	108,160.000	**0.016**	−0.0761
**Pleasure**					
TWBS Total	491.22	562.92	31,246.500	**0.032**	−0.0681
**Need to see morbid things**					
RSS Total	489.97	624.13	17,855.000	**0.001**	−0.1042
SHS Total	490.72	610.68	18,554.500	**0.020**	−0.0999
HSS Total	492.32	581.65	20,064.000	**0.028**	−0.0696
PVS Total	491.89	589.43	19,659.500	**0.017**	−0.0759

Notes: α = Cronbach’s alpha; *M* = mean; *SD* = standard deviation; MR–mean rank; *U* = Mann–Whitney test; *p* = *p*-value; *r* = rank-biserial correlation. In bold: statistically significant values.

**Table 8:** Variables that contribute to the dark tourism practice index.

	Model 1				Model 2	
	B	EP B	β	B	EP B	β
Gender	0.031	0.015	0.066	0.028	0.012	0.060
Age	0.001	0.001	0.052	0.001	0.000	0.076
Know/know not dark tourism	0.069	0.015	0.147	0.030	0.012	0.064
Curiosity				0.130	0.013	0.280
Need to learn				0.107	0.015	0.217
Need to understand				0.107	0.014	0.229
Pleasure				0.108	0.021	0.128
R^2^ (R^2^ Adj.)	0.027 (0.024)			0.385 (0.381)		
F for change in R^2^	9.291 **				143.202 **	

Notes: R^2^ = R squared; R^2^ Adj. = R squared adjusted; B = unstandardized regression coefficients; EP B = unstandardized error of B; β = standardized regression coefficients; ** *p* < 0.001.

## Data Availability

Datasets are available upon request to the authors.
